# Scenario-based supported interventions for moral injury and posttraumatic stress disorder: Data report of film and television references for use with uniformed professionals

**DOI:** 10.3389/fpsyt.2022.917248

**Published:** 2022-09-08

**Authors:** Suzette Brémault-Phillips, Katherine S. Bright, Andrew Phillips, Eric Vermetten

**Affiliations:** ^1^Department of Occupational Therapy, Faculty of Rehabilitation Medicine, University of Alberta, Edmonton, AB, Canada; ^2^HiMARC, Faculty of Rehabilitation Medicine, University of Alberta, Edmonton, AB, Canada; ^3^Department of Community Health Sciences, Cumming School of Medicine, University of Calgary, Calgary, AB, Canada; ^4^Department Psychiatry, Leiden University Medical Center, Leiden, Netherlands

**Keywords:** scenario-based supported interventions, moral injury, PTSD, films, television, uniformed professionals

## Introduction

Uniformed Professionals (UPs), including military members, public safety personnel, and essential service providers, operate in increasingly fast-paced, unpredictable, complex and ambiguous environments. Situations arising in these contexts often require UPs to make prompt decisions and act rapidly to protect themselves and others. While their decision-making is informed by a values-based framework, code of conduct, implicit and explicit duties, and standards of practice, moral dilemmas that arise in the course of service can challenge their values and beliefs. Moral dilemmas are a special class of moral decisions in which (i) there is a conflict between at least two core values/obligations (loyalty, obedience, respect for life); (ii) acting in a way that is consistent with one underlying value means failing to fulfill the other(s); (iii) harm will occur regardless of the option chosen; and (iv) a decision is inescapable and inevitable; some action must be taken (1). In some cases, moral dilemmas can contribute to mental health problems such as PTSD, depression, anxiety, and moral injury (MI).

MI is a psychological and spiritual injury that arises as a result of exposure to a potentially morally injurious event (PMIE), including participating in, witnessing, or failing to prevent an act(s) that transgresses core beliefs (2). Guilt, shame, anger, betrayal, powerlessness, and suicidal ideation are commonly associated with MI. UPs can experience various types of shame and guilt associated with perceived moral transgressions including dishonesty, harm to others, injustice, violation of trust, failure to care, or lack of self-control. They can also experience survivor guilt, guilt over acts of omission or commission, or guilt about thoughts/feelings. Emerging themes in the field of MI include betrayal (e.g., leadership failures, betrayal by peers, failure to live up to one's own moral standards, betrayal by trusted civilians), disproportionate violence (e.g., acts of revenge, mistreatment of combatants), and incidents involving civilians (e.g., harm caused, assault, destruction of property). Moral transgressions associated with violence in service environments (e.g., sexual trauma, friendly fire, “fragging” (i.e., the deliberate or attempted killing of a soldier by a fellow soldier) have also been the focus of significant consideration. These are all difficult to speak about in advance of and following exposure to PMIEs.

Novel approaches and solutions are needed prior to and following exposure to PMIEs to minimize their impact and address PTSD and MI that may result. Such approaches necessitate recognition of moral issues and the development and practice of moral awareness. This requires systematic and continuous interventions focused on enhancing moral reasoning and judgment, and instilling values such as integrity, moral courage, professionalism, and responsibility. We propose that scenario-based supported interventions (SBSIs) that use movie and television references are a novel and promising approach to both stimulate a discourse on PMIEs, PTSD and MI, and support a range of MI interventions including primary prevention, “psychological first aid” training or intervention (3, 4), and individual and group-oriented treatment interventions (5, 6).

SBSIs, informed by moral and ethical training and cognitive-based models, have three substantive objectives: (i) increasing moral awareness, understanding moral dimensions, and recognizing moral implications of their decisions; (ii) exercising moral judgment, recognizing different and at times competing cultural moral systems, and identifying an appropriate understanding of their role in the situation and their potential responses, and (iii) increasing confidence and mastery of managing morally complex situations (7). Importantly, Thompson et al. (8) advocated that scenarios used in primary prevention should be morally ambiguous or complex so that UPs are able to “*confront the absence of “right” answers,... [and understand that] they may not [always] be able to resolve the dilemma, solve the problem, or “do the right thing*”” (p. 279), as there are times when this may be the case in operations (8). We propose that there is a fourth objective: (iv) providing a reflective mirror - where the mirror is a metaphor for the reflective practitioner and one's experiences that are shared among peers through a common language.

SBSIs can be used in psychoeducational classroom settings and therapeutic contexts. The benefits of integrating movie and television references in UP populations into leader-led discussions during professional military education (PME) was first explored by Thompson et al. (8), followed by Warner et al. (9), and Thompson and Jetly (10). Thompson et al. (8) argued that PME provides the time for critical thinking skills to analyze moral challenges (such as contempt, anger, disgust, shame, guilt, awe, honor, elevation, pride), using three key psychoeducational processes: (i) situational reconstruction, in which individuals revisit the experience in order to gain perspective; (ii) focusing, in which individuals explore their moral emotions and physical reactions to the event; as well as (iii) compensatory self-improvement, in which individuals envision what actions they can now take to develop confidence in their ability to take future action [also see de Graaff et al. (11)]. While the video clips and material provided a novel technique to assist leaders with framing the context of the discussion, retaining soldiers' attention, and focusing it on key training concepts, the greatest impact of the program came from the chain-teaching format: “*The brief video clips provided a framework for discussion of topics relevant to the day-to-day scenarios that these soldiers were encountering, sometimes including ambiguous and difficult ethical dilemmas. The chain teaching provided a method for unit leaders to give guidance on how they expected their subordinates to respond to ethically challenging situations and also allowed for direct discussion between participants about situations they had actually encountered in their work*.” (9, p. 922). A recent scientific review of the effectiveness of a training program for military leaders using SBSIs noted significant reductions in soldier mistreatment of noncombatants and simultaneous improvement in soldiers' ethical attitudes (9). Peer insights and support, mentor supervision, and access to mental health professionals within resident PME can not only allow UPs to “*prepare themselves for the morally traumatic situations they may experience during future deployments, and learn how to prepare their colleagues to do the same”* (8, p. 278), but “*create an environment in which they can process past PMIEs*” (8, p. 278).

Movie vignettes and television clips have also been used as cinematherapy to address PMIEs and MI. In addition to cinematherapy, SBSIs using movie and television clips may be helpful in a variety of psychoeducational contexts and include evidence-based methods such as group movie therapy, art therapy, and bibliotherapy. Four distinct but connected stages of self-development can be facilitated through cinematherapy: (i) identification, (ii) emotional release, (iii) insight, and (iv) universalization (12, 13). A teacher or clinician can use these stages to stimulate and structure discussions for prevention through treatment of MI. During the identification stage, individuals see a commonality, similarity, and/or connection with the character and/or situation. This stage offers an opportunity for examination of the behaviors and motives of the characters and self-exploration. In the second stage, individuals can work through a problem and emotions that surface, and release emotions and tensions. In the third stage, by understanding the behaviors and motivations of a character, individuals can empathize with and develop better awareness and understanding of issues and situations within their own lives. In the universalization stage, individuals recognize that others have similar experiences and difficulties. Individuals can experience an increased sense of community and reduced sense of isolation, aloneness, and shame or guilt (14). As an SBSI, cinematherapy can foster critical thinking skills. *Via* situational reconstruction, moral challenges can be experienced, physical reactions/responses to these events can be explored, and actions can be envisioned that help build confidence in future decision-making and action-taking measures.

UP leaders are among those exploring innovative approaches to address PMIEs in pre/post-deployment training. The research by Thompson et al. (8) was the first of its kind to encourage the use of SBSIs in UP populations. Our goal was to establish and describe a dataset of relevant movie and television references for use as SBSIs with UPs prior to exposure to PMIEs or in the course of treatment for MI. Development of the dataset was informed by Ge et al. (15), who created an expanded database of emotional film clips for use in treatment with individuals diagnosed with schizophrenia.

## Methods

An environmental scan of popular cinema and television shows was conducted between November 2019 and April 2022 with the aim of isolating relevant and accessible English language movies and television episodes produced between 1930 to 2022 for inclusion in the dataset. This included searches of Internet film databases (e.g., imdb.com, wingclips.com, trakt.tv, ranker.com), online movie scripts (e.g., script-o-rama.com, quodb.com, scripts-onscreen.com, dailyscript.com), screenplays (e.g., sfy.ru, moviescriptsandscreenplays.com, scripts.tv-calling.com), review articles of films about trauma and PTSD, and film and television review sites. Keywords used in the searches included post-traumatic stress (PTSD), battle fatigue, trauma, anguish, stress, shellshock, anxiety, depression, isolation, shame, and grief. Screening and selection of movies and television episodes was conducted by a research team member (AP) based on a search of films by theme and title, review of film scripts, and screening of movies and television episodes to determine clip relevance, ecological validity, relatability by selected populations, popularity, length, viewability, and depiction of a growth-oriented character arc (i.e., exposure to a PMIE or potentially traumatic experience, followed by potential or obvious healing/recovery). Excluded were low-budget schlock horror films (e.g., overly-sensationalized and excessively violent, gory clips or those depicting victimization, abuse or exploitation), and films wherein characters did not exhibit a growth-oriented character arc in a manner easily depicted in a video clip.

A database protocol was developed and a spreadsheet prepared to capture study findings. A preliminary searchable dataset was constructed including descriptive information/categories to facilitate selection of clips appropriate for psychoeducational or therapeutic use. The clips included were limited in length as per fair dealing/copyright regulations, with linkage to online digital delivery platforms (e.g., Films-on-Demand, Criterion-On-Demand) being explored.

## Dataset

### Data collection protocol

**Figure 1:**
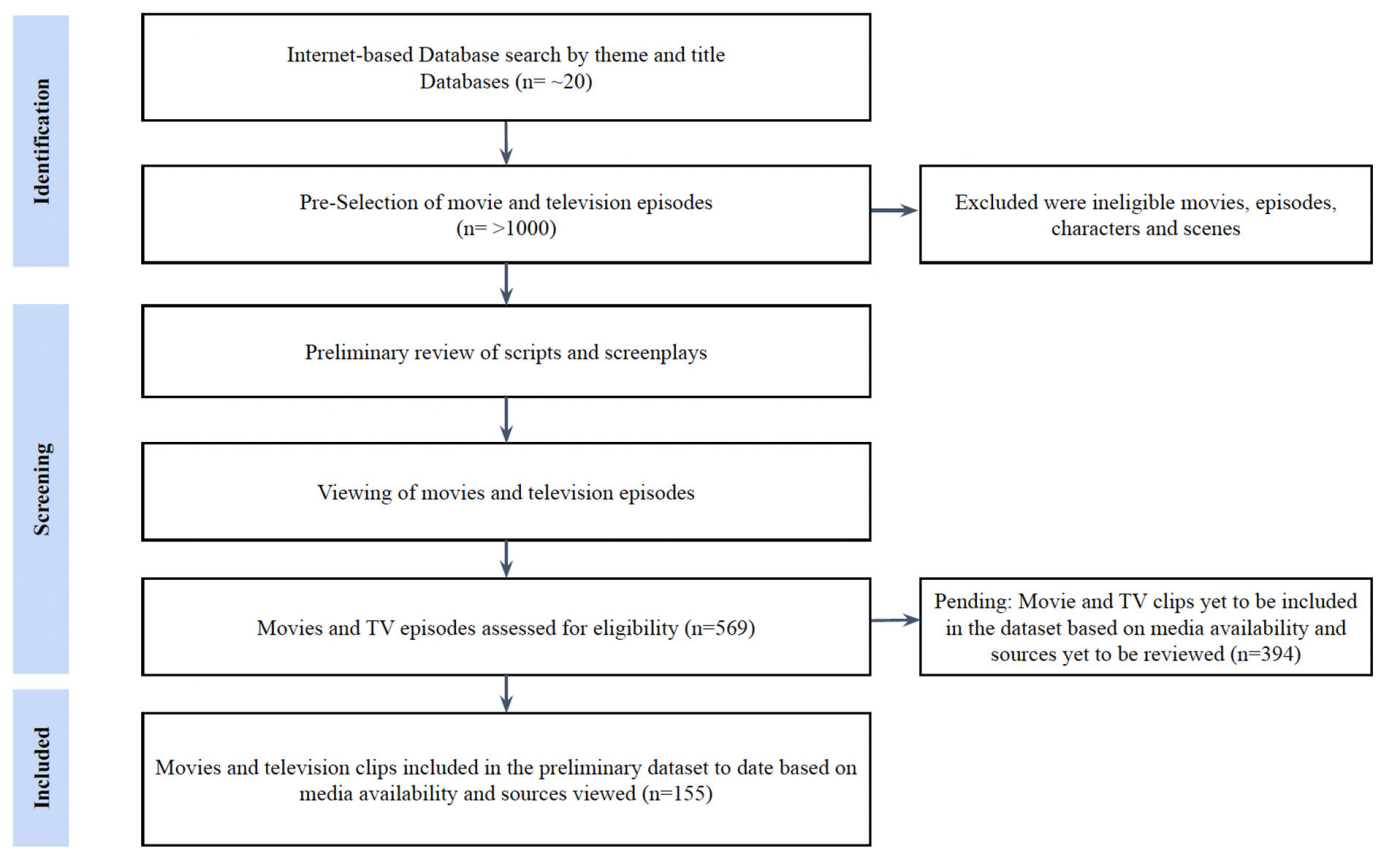
Diagram of workflow steps (data collection and primary curation) for creating the SBSI Dataset.

More than one thousand movies and television episodes were selected. After reviewing scripts and screenplays, viewing films and television episodes, and screening by inclusion/exclusion criteria, a total of 569 movie and television show references were identified. Findings were compiled by AP into an initial searchable dataset containing 155 video clips for creating the SBSI dataset. Clips from movie and TV episodes (*n* = 394) are yet to be reviewed and included in the dataset based on media availability and sources [see Figure 1: Diagram showing the workflow steps (data collection and primary curation)].

Clips were cataloged by theme, population (i.e., Emergency Medical Services (EMS), Health Care, Fire, Police, Military, Pseudo-Military), trauma symptom/traits (i.e., avoidance, depression, emotional dysregulation, flashbacks, guilt/shame, nightmares, self-harm) and PTSIs (i.e., substance misuse, institutional betrayal, sanctuary trauma, military sexual trauma, burnout, complex grief, PTSD complex) for use by educators, mental health professionals and researchers. Movie title, type, release date, director, production company, clip length, relevance, intensity, and brief descriptions were also included. The dataset has been developed for ease of access by potential users and within various contexts (e.g., psychoeducation, peer support, family support, social support, and psychotherapy/cinematherapy). This effort resulted in a unique dataset that can be used at various stages of intervention, from primary prevention through treatment.

### Dataset description and structure

The dataset consists of a range of information. This includes the full name of the movie or television show, release date, director, production company, film length, clip time codes, and a description. The type of scene/experience is also included (e.g., army training, combat trauma, moral injury, noncombat PTSD, sexual assault, trauma, childhood trauma, veterans (Korean, WW1, WW2, OED/OIF/OND, Vietnam, Indigenous, pseudo-soldiers, Civil War, Russian), civilians, documentary, parody, and anime). Each clip is rated for relevance on a Likert scale (1 being of little relevance to 10 being most relevant) and intensity/potentially triggering content (1 being mild and 10 being disturbing). External links to the clips are also applied. An information section includes as a brief description of the clip's content. The dataset also includes a variety of symptoms/traits (including avoidance, depression, emotional dysregulation, flashbacks, guilt/shame, nightmares, self-harm), and lists a number of post-traumatic stress injuries (PTSIs) (including substance misuse, institutional betrayal, sanctuary trauma, military sexual trauma, burnout, complex grief, and complex PTSD). The specific population for whom the video clip is relevant is noted (e.g., peer support, military, police, fire, health care, EMS, and civilian). Lastly, there is a section in the dataset that describes where the clip may be helpful as an SBSI including psychoeducation, psychotherapy, and spiritual, peer, social, and family support (see Table 1 for Dataset structure).

**Table 1:** Dataset structure.

**General information**	**Symptoms/traits**	**Post-traumatic stress injuries**	**Populations**	**Interventions**
Media Title	Avoidance	PTSD	Pseudo-Military	Psychoeducation
Release Date	Depression	Moral Injury	Military	Psychotherapy
Director	Emotional Dysregulation	Complex Grief	Police	Spiritual Support
Production Company	Flashbacks	Institutional Betrayal	Fire	Peer Support
Length	Guilt, Shame	Substance Misuse	Health Care	Family Support
Film or TV Show Type	Nightmares	Sanctuary Trauma	EMS	Social Support
Relevance	Self-Harm	Military Sexual Trauma	Civilian	
Intensity		Burnout		
Viewable Clip				
External Resources				
Information				

### Interpretation and use

SBSIs are a novel means of using popular culture movie vignettes and television clip references that can facilitate self-reflection and stimulate discourse of salient topics around PMIEs, PTSD and MI. Use of the popular culture references in this catalog offers a novel approach for interventions, from primary prevention through therapeutic interventions. The creation of this work adds a readily available and searchable database, “*tools at one's fingertips*,” that makes it easier to locate and use relevant clips for engaging in training or therapy. This evolving dataset will be made available and disseminated in compliance with copyright regulations.

SBSIs and this database can be used in individual or group contexts. In individual settings, this could be facilitated using Head Mounted Displays where the participant can have an immersive personalized experience of the movie or television clip. In a group setting, these clips can be viewed together, creating a shared experience and stimulating a discourse around experiences and topics that are otherwise difficult to discuss. The metaphor of a mirror can be used to guide the tensions between the individual's reflective listening process and his or her inner experience while reflecting. The conceptualization of reflective listening constitutes a dialectical shift that opens a different approach to the problem of the “tain or back surface,” eventually concluding in an interactional formulation of reflection as the provision of tentative understandings (16). These understandings are designed to be amended in response to feedback. The reflective mirror, in the context of psychoeducational training, specifically SBSIs, provides the opportunity for enhancing reflexivity and reflective thinking during training. SBSIs can also be used in relation to sensitive times in relation to deployment.

#### Pre-deployment

For purposes of prevention and health promotion, resources from this dataset can be integrated in psychoeducational classroom discussions preparing individuals for experiences they may face in service contexts. The use of SBSIs provides the time for critical thinking skills and self-awareness in experiencing moral challenges *via* situational reconstruction, and exploring physical reactions/responses to these events (17). They may also contribute to envisioning what actions will further develop the confidence of UPs in future decision-making and action-taking measures.

#### Post-deployment

This database can also be used in critical incident debriefing, or therapeutic contexts and psychoeducation to enable individuals to process exposures to PMIEs and facilitate recovery. SBSIs, with the use of film clips, facilitates the reconstruction of past experiences and provides UPs with the opportunity to explore how events may be different from what they previously experienced. The use of videos in therapy, cinematherapy, has been shown to facilitate self-awareness and development, connection with common experiences, elicitation and release of emotions, gaining of insight, and awareness that they are not alone (12–14). Ultimately, SBSIs that use film clips provide opportunities for self-disclosure and discussion which may result in increased self-esteem, positive coping mechanisms, and decision-making skills (18). As such, SBSIs are important in developing a strong sense of self, and buffering against adversity and crisis, resulting in a willingness to engage in action-taking measures (19).

## Conclusion

UP leaders are incorporating a variety of training methods into their pre- post-deployment training. A description of a searchable database of movie and television references focused on PMIEs, PTSD, and MI is presented for use as SBSIs with UPs. Used in psychoeducational and therapeutic contexts, SBSIs may provide a common shared language. SBSIs may provide a common shared language, and means of normalizing and reducing stigma associated with PMIEs. Association and identification with characters in movie and television clips may facilitate empathy while simultaneously increasing awareness, understanding and reflection and addressing unresolved feelings such as grief, loss, shame and guilt associated with MI. In a group setting, movie clips can provide the medium to discuss these sensitive situations and emotions. As the use of SBSIs with UPs, however, is in its infancy and is not standard protocol, training for UP leaders and therapists will yet need to be developed to inform how to most appropriately and effectively incorporate such interventions if they are to have any effect on reducing the impact of PMIEs on UPs or support treatment for moral injury. The dataset will continue to evolve, be updated and be made available and disseminated in compliance with copyright regulations. We look forward to conducting a proof-of-concept study to initially explore the feasibility and acceptability of this evolving dataset into pre/post-training and therapy, and the feasibility and acceptability among military members and public safety personnel and UP leaders providing training and therapeutic interventions.

## Data availability statement

The raw data supporting the conclusions of this article will be made available by the authors, without undue reservation and in compliance with copyright regulations.

## Author contributions

AP contributed to the primary data search strategies, data collection, and development of the database. SB-P and EV contributed to the project design, article preparation, and overall supervision. All authors contributed to the article and approved the submitted version.

## Conflict of interest

The authors declare that the research was conducted in the absence of any commercial or financial relationships that could be construed as a potential conflict of interest.

## Publisher's note

All claims expressed in this article are solely those of the authors and do not necessarily represent those of their affiliated organizations, or those of the publisher, the editors and the reviewers. Any product that may be evaluated in this article, or claim that may be made by its manufacturer, is not guaranteed or endorsed by the publisher.
